# An emerging and promising anticancer strategy: colonization of engineered *Staphylococcus epidermidis*

**DOI:** 10.1002/mco2.467

**Published:** 2024-01-12

**Authors:** Yanyan Liu, Yulong Cai

**Affiliations:** ^1^ Laboratory of Aging Research and Cancer Drug Target, Department of Biotherapy and Cancer Center, National Clinical Research Center for Geriatrics, West China Hospital Sichuan University Chengdu Sichuan China; ^2^ Division of Biliary Tract Surgery, Department of General Surgery West China Hospital Sichuan University Chengdu Sichuan China

1

A recent study published in the journal *Science* by Chen et al.^1^ proposed an emerging and promising anticancer strategy that utilizes engineered skin bacteria to treat or prevent melanoma. The research shed light on the potential of microbiome to modulate host immunity and offered novel insights into the application of commensal microorganisms in human diseases.

After a long period of coevolution, humans live in symbiosis with a variety of microorganisms, including gut microbes, skin symbionts, fungi, and viruses. Among them, skin microorganisms are a complex group containing bacteria, fungi, and viruses, which are involved in constituting the first protective barrier for humans.^2^ It is worth mentioning that symbiotic bacteria have two roles that should not be overlooked: they are involved in the regulation of the local or systemic immune environment, and bacterial proteins can induce specific immune responses.^3^ For instance, in 2012, a study reported that the skin microbiota can actively control local inflammatory responses and regulate resident T lymphocyte function.^4^ Moreover, utilizing the immune function of the body is one of the significant tumor therapies. Based on these understandings, we would raise the question, could these interactions be used for antitumor therapy? Prof. Michael A. Fischbach's group from Stanford University explored this question and gave us the answer (Figure 1).^1^

**FIGURE 1:**
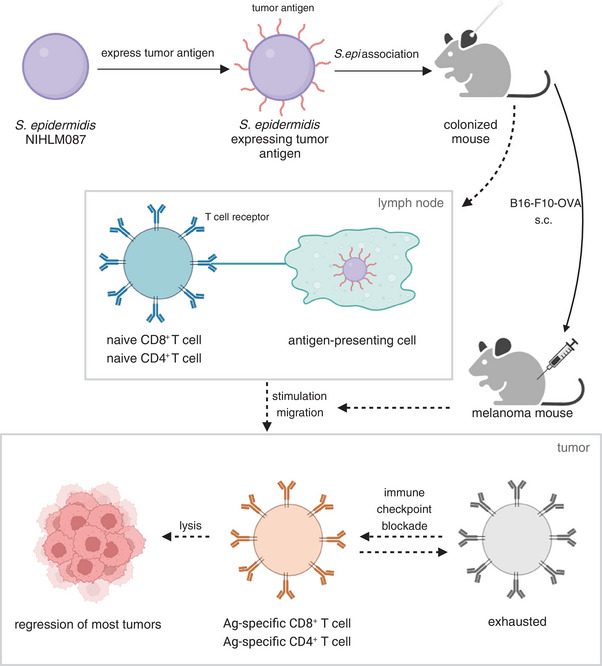
Engineered *S. epidermidis* strains show tumor regression and synergize with immune checkpoint blockade. Engineered *S. epidermidis* strains colonize the skin and induce antigen‐presenting cells to stimulate antigen‐specific T cells, which migrate to the tumor and slow tumor growth. Immune checkpoint blockade synergizes excellently with engineered *S. epidermidis*. The figure is created by BioRender. Red lines represent antigenic peptides. Solid arrows indicate research steps and dashed arrows indicate the cellular immune processes in mice after immunization with engineered modified *S. epidermidis*.

*Staphylococcus epidermidis* is a ubiquitous bacterium that colonizes the human skin and plays a significant role in the barrier development, maintenance of homeostasis and controlling of opportunistic pathogens.^5^ First, the researchers expressed the tumor antigens in *S. epidermidis* and found that skin commensal‐induced T cells were able to recognize tumors specifically. This allowed for the observation of whether these T cells were able to infiltrate the tumor and participate in a cytotoxic attack on the tumor. To be specific, they constructed 13 mutant strains of *S. epidermidis* using a specialized DNA‐importing system with an *Escherichia coli* DC10B passaging plasmid, heat shock, and hyperosmolar sorbitol. Next, *S. epidermidis* NIHLM087 was selected to express unnatural antigens and ovalbumin (OVA) was used as a model antigen. Three groups of strains with different antigen properties and localization were constructed. *S. epidermidis* expressing two antigenic peptides from OVA, OT‐I and OT‐II, could stimulate OVA‐specific CD8^+^ or CD4^+^ T cells, respectively. There are three different antigenic localizations, including cytoplasmic (represented by “c”), covalently bound to the cell wall (represented by “w”), and secreted extracellularly (represented by “s”). Through colonization with *S. epidermidis* expressing either unrelated or related antigens before subcutaneous injection of B16‐F0‐OVA (an OVA‐expressing melanoma cell line) into mice, it was discovered that *S. epidermidis* has antitumor activity only in the presence of OVA, indicating that OVA‐specific T cells are required for the antitumor effect. Additionally, the absence of antitumor responses in heat‐inactivated *S. epi*‐OVApep (a combination of *S. epi*‐wOT‐I and *S. epi*‐sOT‐II) suggested that the engineered bacteria not only act as antigens and adjuvants, but their ability to survive, occupy specialized ecological niches, and prolonged antigen exposure are probably necessary for immune stimulation responses. The antitumor activity was lost when mice were not related to both *S. epi*‐wOT‐I and *S. epi*‐sOT‐II combinations, suggesting that the necessary conditions for the antitumor effect are OVA‐specific CD8^+^ T and CD4^+^ T cells.

Besides in situ and near colonization, they examined whether the engineered *S. epidermidis*‐induced T cells could migrate to other tissues. If so, whether the displacement of microbial colonists that can lead to sepsis is a potential concern. By using B16‐F10, a highly aggressive model of metastatic melanoma, the study showed that the antitumor effect of *S. epi*‐OVA is not limited to the skin and subcutaneous tissues. Colonization with *S. epi*‐OVA significantly impeded the growth of lung metastatic melanoma cells. *S. epi*‐OVA‐induced T cells limited aggressive tumor growth even as monotherapy, and meanwhile, the treatment is mild and does not cause any immune side effects, which obviates the previous concern.

Encouraged by these positive results, the researchers proceeded to explore whether the engineered *S. epidermidis* expressing other tumor antigens could induce specific T cell responses, further exerting antitumor effects. They then engineered *S. epidermidis* to express two neoantigenic peptides from B16‐F10 melanoma (termed *S. epi*‐neoAg). Prophylactic colonization of *S. epi*‐neoAg effectively suppressed subsequent metastatic tumor growth. Besides, they evaluated the therapeutic effect of engineered *S. epidermidis* by colonizing *S. epi‐*OVApep at 3 days after intravenous inoculation of B16‐F10‐OVA (a melanoma cell line expressing OVA) in the metastatic melanoma model. The inhibition of tumor growth in the treatment model was comparable to that in the prevention model, and OVA‐specific CD8^+^ T cells in the lung and spleen increased. The result supports the importance of *S. epi*‐OVApep‐induced CD8^+^ T cells in controlling melanoma metastasis. In addition, although *S. epi*‐OVA‐induced and tumor‐induced T cells have the same antigen specificity, they exhibit different functions. The composition of OVA‐specific CD8^+^ T cell population was altered, with the majority of central memory T cells in the control group and effector T cells and effector memory T cells in the *S. epi*‐OVApep group. Nevertheless, the effector T and effector memory T cells could be exhausted in the tumor environment, and the tumor‐infiltrating lymphocytes induced by *S. epi*‐OVA highly expressed the marker of exhaustion, programmed cell death protein‐1. Consequently, they further found that the combination of engineered *S. epidermidis* with immune checkpoint inhibitors could produce a durable and effective antitumor response to treat established melanoma without significant adverse effects, implying the broad application prospect of antitumor therapy exerted by engineered *S. epidermidis*.

This study has demonstrated the antigen‐specific antitumor potential of engineered *S. epidermidis*, offering promising prospects for future cancer therapeutics. Moreover, it opens up possibilities for incorporating antigen expression into other commensal microorganisms to induce a broad immune response against various antigens. This compelling study suggests an early but encouraging anticancer strategy. However, it is crucial to acknowledge that *S. epidermidis* is commonly encountered as an opportunistic pathogen.^5^ Since *S. epidermidis* also inhabits the gut, antigen‐expressing *S. epidermidis* strains in the skin may lead to contamination of other nonskin microorganisms in vivo, triggering systemic antimicrobial immune responses. Also, engineered commensals may also induce tolerance responses, particularly in certain autoimmune diseases, allergic diseases, and chronic inflammatory conditions. Meanwhile, there has been more research on the relationship between gut microbes and immune function, and a lack of research on skin symbiotic bacteria, which also play an essential role in immune function. Therefore, a large number of in‐depth studies are warranted. In addition, research in multiple classical cancer models would be beneficial in expanding the application of engineered bacteria. Since the structure and function of the mouse and human body are quite different, additional animal model studies are imperative. Moreover, thorough research on the interaction between the commensal microbiota and the host is beneficial to enhance the safety and efficacy of this therapy for a better clinical application.

## AUTHOR CONTRIBUTION

Y. C. conceptualized the research highlight and Y. L. wrote the manuscript and drew the figure. Both the authors have read and approved the final manuscript.

## CONFLICT OF INTEREST STATEMENT

The authors declare no conflict of interests.

## FUNDING INFORMATION

This work has no relevant funding.

## ETHICS STATEMENT

No ethics approval was required for this review that did not involve patients or patient data.

## Data Availability

Not applicable.
